# An evidence-based warfarin management protocol reduces surgical delay in hip fracture patients

**DOI:** 10.1007/s10195-015-0351-1

**Published:** 2015-05-15

**Authors:** M. Diament, W. G. P. Eardley

**Affiliations:** Department of Trauma and Orthopaedics, James Cook University Hospital, Middlesbrough, UK

We read with interest the work of Ahmed et al. [1] regarding anticoagulation reversal in the hip fracture population. Whilst congratulating the authors on the clarity of their protocol, we would like, if we may, to highlight a number of areas we feel require further explanation.

Hip fracture care in the United Kingdom (UK) has been transformed in recent years. Care is now optimised through best practice tariffs (BPT), a financial incentive scheme that advises, amongst other domains, that patients with a hip fracture undergo surgery within 36 h of admission [2]. Ahmed et al. work within this system in the UK. Time to surgery is a major driver of care and is the subject of contemporary literature [3]. It is concerning, therefore, that there is no mention of the performance of their population in terms of the biggest outcome assessment used in UK trauma care. This omission is puzzling. Also, despite current guidelines advising that time to theatre should be within 36 h, the authors ignored this and chose 48 h instead.

Having used a 48-h outcome, Ahmed et al. state that 80 % of their cases achieved surgery within this time frame. The raw data, however, do not support this. The authors state that 21 of their patients required more than one dose of IVK. Following their flow chart, these 21 patients would, once in receipt of their second dose of IVK, have waited a further 24 h before having their INR rechecked. Put simply, 21 of 40 patients waited at least 48 h before being in a position to be considered for surgery, never mind actually having an operation. With over 50 % of patients unable to reach a decision point on fitness to operate until at least 48 h, how can they conclude that 80 % of the same population had an operation within the same time period?

There are a number of other issues with this study. There were apparently no patients in either group that were cancelled or delayed to theatre for reasons other than warfarin reversal. Ahmed et al. indicate that this is the case, as there is no disclosure of comorbidities (unwell patients requiring optimisation prior to surgery) or logistic delays (inability to access the trauma theatre due to caseload). These two elements are the biggest challenges to timely hip fracture care in clinical practice.

The wider readership and particularly those of us practising in the United Kingdom would benefit from some clarity on the above points. This would help hip fracture units decide on whether or not to adopt this protocol in light of the data discrepancies above and the impact this may have on BPT revenue.
